# Injury-induced cooperation of InhibinβA and JunB is essential for cell proliferation in *Xenopus* tadpole tail regeneration

**DOI:** 10.1038/s41598-024-54280-w

**Published:** 2024-02-14

**Authors:** Makoto Nakamura, Tatsuya Kyoda, Hitoshi Yoshida, Kimiko Takebayashi-Suzuki, Ryota Koike, Eri Takahashi, Yuka Moriyama, Marcin Wlizla, Marko E. Horb, Atsushi Suzuki

**Affiliations:** 1 Amphibian Research Center, Graduate School of Integrated Sciences for Life, Hiroshima University, 1-3-1 Kagamiyama, Higashi-Hiroshima, Hiroshima 739-8526 Japan; 2 National Xenopus Resource and Eugene Bell Center for Regenerative Biology and Tissue Engineering, Marine Biological Laboratory, Woods Hole, MA 02543 USA; 3 Present Address: Cardiovascular Research Institute, University of California San Francisco, 555 Mission Bay Boulevard South, San Francisco, CA 94158 USA; 4 Present Address: Embryology, Charles River Laboratories, Wilmington, MA 01887 USA

**Keywords:** InhibinβA, JunB, FGF, *Xenopus* tail regeneration, Regenerative cell proliferation, Developmental biology, Genetics

## Abstract

In animal species that have the capability of regenerating tissues and limbs, cell proliferation is enhanced after wound healing and is essential for the reconstruction of injured tissue. Although the ability to induce cell proliferation is a common feature of such species, the molecular mechanisms that regulate the transition from wound healing to regenerative cell proliferation remain unclear. Here, we show that upon injury, InhibinβA and JunB cooperatively function for this transition during *Xenopus* tadpole tail regeneration. We found that the expression of *inhibin subunit beta A* (*inhba*) and *junB proto-oncogene* (*junb*) is induced by injury-activated TGF-β/Smad and MEK/ERK signaling in regenerating tails. Similarly to *junb* knockout (KO) tadpoles, *inhba* KO tadpoles show a delay in tail regeneration, and *inhba*/*junb* double KO (DKO) tadpoles exhibit severe impairment of tail regeneration compared with either *inhba* KO or *junb* KO tadpoles. Importantly, this impairment is associated with a significant reduction of cell proliferation in regenerating tissue. Moreover, JunB regulates tail regeneration via FGF signaling, while InhibinβA likely acts through different mechanisms. These results demonstrate that the cooperation of injury-induced InhibinβA and JunB is critical for regenerative cell proliferation, which is necessary for re-outgrowth of regenerating *Xenopus* tadpole tails.

## Introduction

Amphibians, such as *Xenopus*, can regenerate lost tissues after injury; this high capacity for regeneration has attracted attention for its potential applications in regenerative medicine^1–3^. Wound healing occurs as a cellular response to injury, and in regenerative animals, proliferation of regenerating cells is subsequently enhanced to restore lost tissue. It has been reported that inhibition of cell cycle progression after wound healing causes a severe delay in regeneration^4,5^, indicating that injury-induced cell proliferation is required for successful tissue regeneration. In neonatal mice, cardiomyocyte proliferation increases after cardiac apex resection; however, from one-week-old, the proliferation response does not occur in the mice even though the wound is closed, resulting in the failure of heart regeneration^6^. In adult mice, overexpression of cell cycle regulators after heart damage induces cardiomyocyte proliferation and improves heart function^7^. On the basis of these findings, it appears that injury-induced cell proliferation may be suppressed in non-regenerative animals. Therefore, the transition from wound healing to regenerative cell proliferation is a crucial process for tissue regeneration.

*Xenopus* tadpole tail regeneration is divided into two main phases: wound healing and proliferation. During the wound healing phase, Smad2/3 (signal transducers of TGF-β signaling) and ERK are known to be activated in response to injury, and these signaling factors play important roles in tissue regeneration^4,8^. After wound healing, tail regeneration proceeds to the proliferation phase, in which formation of the regeneration bud and regenerative outgrowth/cell proliferation occur. In the proliferation phase, morphogenetic signals, such as BMP, Wnt, and FGF, are critical for regenerative cell proliferation; these signals are also found to be important in other regenerative species^3,9–13^. In *Xenopus* tail regeneration, the genetic perturbation of BMP signaling reduces the number of proliferating cells in the regenerating tissue, and the activation of Wnt signaling promotes regenerative cell proliferation^9,10^. FGF acts downstream of BMP and Wnt signaling^9^, and it has been reported that FGF and BMP signaling are involved in cell proliferation during *Xenopus* limb regeneration^14^. Although regulatory factors that function in the wound healing and proliferation phases have been identified, the molecular mechanisms regulating the transition from wound healing to regenerative cell proliferation are not yet well understood.

We have previously shown that expression of *junB proto-oncogene* (*junb*) is induced by injury-activated TGF-β/Smad signaling during the wound healing phase and that JunB positively regulates cell proliferation in *Xenopus tropicalis* tail regeneration^15^. However, regenerative cell proliferation is not completely suppressed in *junb* KO tadpoles, suggesting that additional regulators promote cell proliferation in cooperation with JunB. In this study, we found that expression of both *inhibin subunit beta A* (*inhba*) and *junb* is induced through TGF-β/Smad and MEK/ERK signaling after injury. In addition, CRISPR/Cas9-mediated KO experiments revealed the cooperation of InhibinβA and JunB for regenerative outgrowth/cell proliferation. Interestingly, JunB regulates tail regeneration via FGF signaling, whereas the modulation of outgrowth/cell proliferation by InhibinβA is likely mediated by other mechanisms, acting either in parallel with or downstream of FGF signaling. In summary, the cooperative action of InhibinβA and JunB guides the transition from wound healing to regenerative cell proliferation in *Xenopus* tail regeneration. These findings shed light on the mechanisms underlying injury-induced cell proliferation in regenerative animals.

## Results

### The expression of *inhba* and *junb* is induced by injury-activated signaling

**Figure 1:**
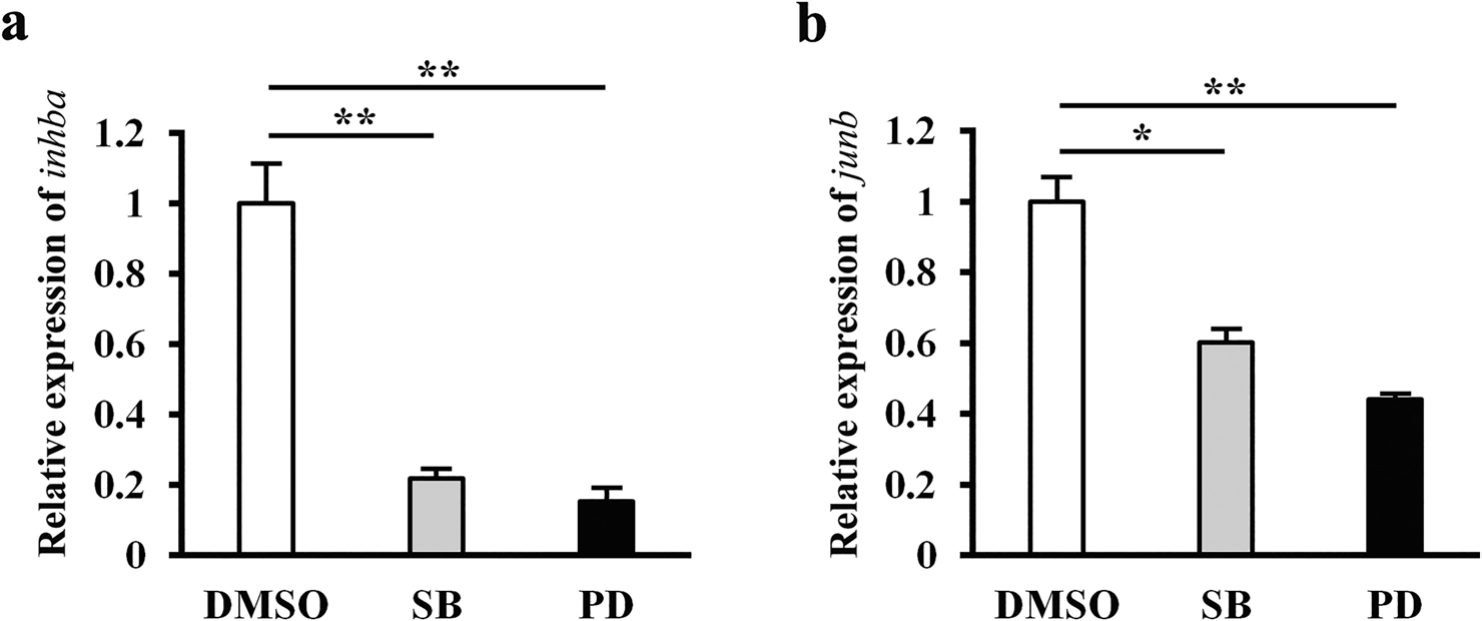
The expression of *inhba* and *junb* is regulated by injury-activated signaling during wound healing. qRT-PCR analysis of *inhba* (**a**) and *junb* (**b**) expression in DMSO (control), SB-505124 (SB), and PD0325901 (PD)-treated tails. The regenerating tails were isolated at 1 and 2 hpa for the expression of *junb* and *inhba*, respectively. The data were normalized against expression of *rps18*, and then by the value of DMSO. *P < 0.05, **P < 0.01.

Although injury-induced JunB is important for regenerative outgrowth/cell proliferation in the *Xenopus* tadpole tail, a CRISPR/Cas9-mediated *junb* KO in F0 or compound heterozygous tadpoles is not sufficient to block tail regeneration^15^. This prompted us to search for regulators that cooperate with JunB for regenerative outgrowth/cell proliferation. Inhibition of TGF-β signaling in the regenerating tail after wound healing impairs cell proliferation in the regeneration bud^4^; thus, in addition to a TGF-β/Activin family ligand responsible for the wound healing phase, other TGF-β/Activin family ligands possibly work together with JunB to promote regeneration during the proliferation phase. To identify candidate TGF-β/Activin family ligands that cooperate with JunB for regenerative cell proliferation, we examined the levels of expression of ligands (Tgfβs, Inhibins, Nodals, Gdfs, and Myostatins) during *X. tropicalis* tadpole tail regeneration using published RNA-seq data^16^ (Supplementary Figure 1). Three genes belonging to the TGF-β/Activin family, namely *inhba*, *tgfb1*, and *tgfb2*, were identified as being highly expressed during the wound healing phase [~ 6 h post-amputation (hpa)] as is *junb*. However, only expression of *inhba* is upregulated after injury (Supplementary Figure 2). Since Smad2/3 and ERK are activated immediately after tail amputation^4,8^, we analyzed whether the injury-mediated increase in expression of *inhba* and *junb* is regulated by TGF-β/Smad and MEK/ERK signaling. As shown in Fig. 1, expression of *inhba* and *junb* during wound healing was downregulated by both SB-505124 (SB, TGF-β receptor inhibitor) and PD0325901 (PD, MEK/ERK inhibitor). These results suggest that, in addition to JunB, InhibinβA may also function downstream of injury-activated signaling to promote cell proliferation in *Xenopus* tail regeneration.

As injury-activated TGF-β/Smad and MEK/ERK signaling are important for the induction of *inhba* and *junb* expression, we investigated the relationship between the activation of Smad2/3 and ERK after tail amputation. In contrast to *tgfb1* KO tadpoles^17^, phosphorylated Smad2/3 (pSmad2/3) expression was not downregulated by PD treatment at 2 hpa (Supplementary Figure 3). It has been shown previously that ERK phosphorylation does not require TGF-β/Smad signaling during wound healing after amputation of the *Xenopus* tail^8^. Taken together, these observations suggest that activation of TGF-β/Smad signaling and of MEK/ERK signaling are unlikely to be dependent on each other at the beginning of wound healing but both are important for the coordinated expression of *inhba* and *junb*.

### *inhba* is expressed in the regeneration bud of the tail

**Figure 2:**
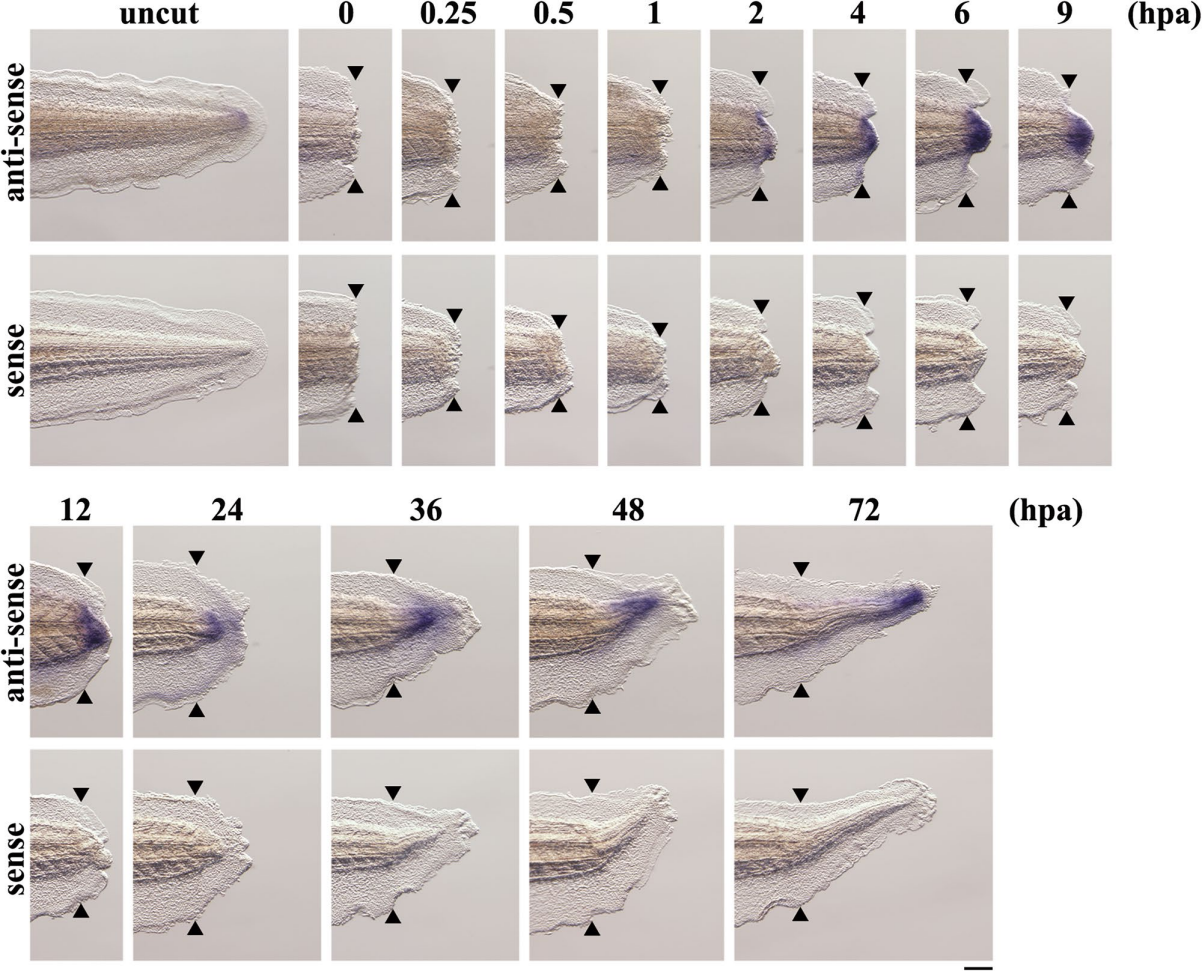
Spatiotemporal expression pattern of *inhba* during *Xenopus* tail regeneration. Whole-mount in situ hybridization of *inhba* in uncut tails and in regenerating tails at 0, 0.25, 0.5, 1, 2, 4, 6, 9, 12, 24, 36, 48 and 72 hpa. Negative control using a sense probe shows no signals. Blue/purple signals indicate the expression of *inhba*. Black arrowheads indicate amputation sites. Scale bar, 200 μm.

We examined the spatiotemporal expression pattern of *inhba* during *Xenopus* tail regeneration using whole-mount in situ hybridization (Fig. 2). A low level of *inhba* expression was observed in the tip of the uncut tail. After amputation, *inhba* expression was not detected until 1 hpa; clear *inhba* expression was observed near the amputation site at 2–4 hpa in the wound healing phase. From 6 hpa, *inhba* transcripts increased in the regenerating tail core tissue at the tip of the amputated tail stump. In the proliferation phase, *inhba* expression was detected in the regeneration bud and the posterior region of regenerating tail at 24 and 36–72 hpa, respectively. Sectioning of tadpoles after in situ hybridization showed that *inhba* was locally expressed in the regenerating mesenchyme, spinal cord, notochord, and epidermis (Supplementary Figure 4); this pattern of expression in regenerating tissue overlapped with that of *junb*. These data indicate that *inhba* is induced by tail amputation and is expressed during regeneration in the *Xenopus* tadpole tail.

### InhibinβA is required for regenerative outgrowth/cell proliferation in cooperation with JunB

**Figure 3:**
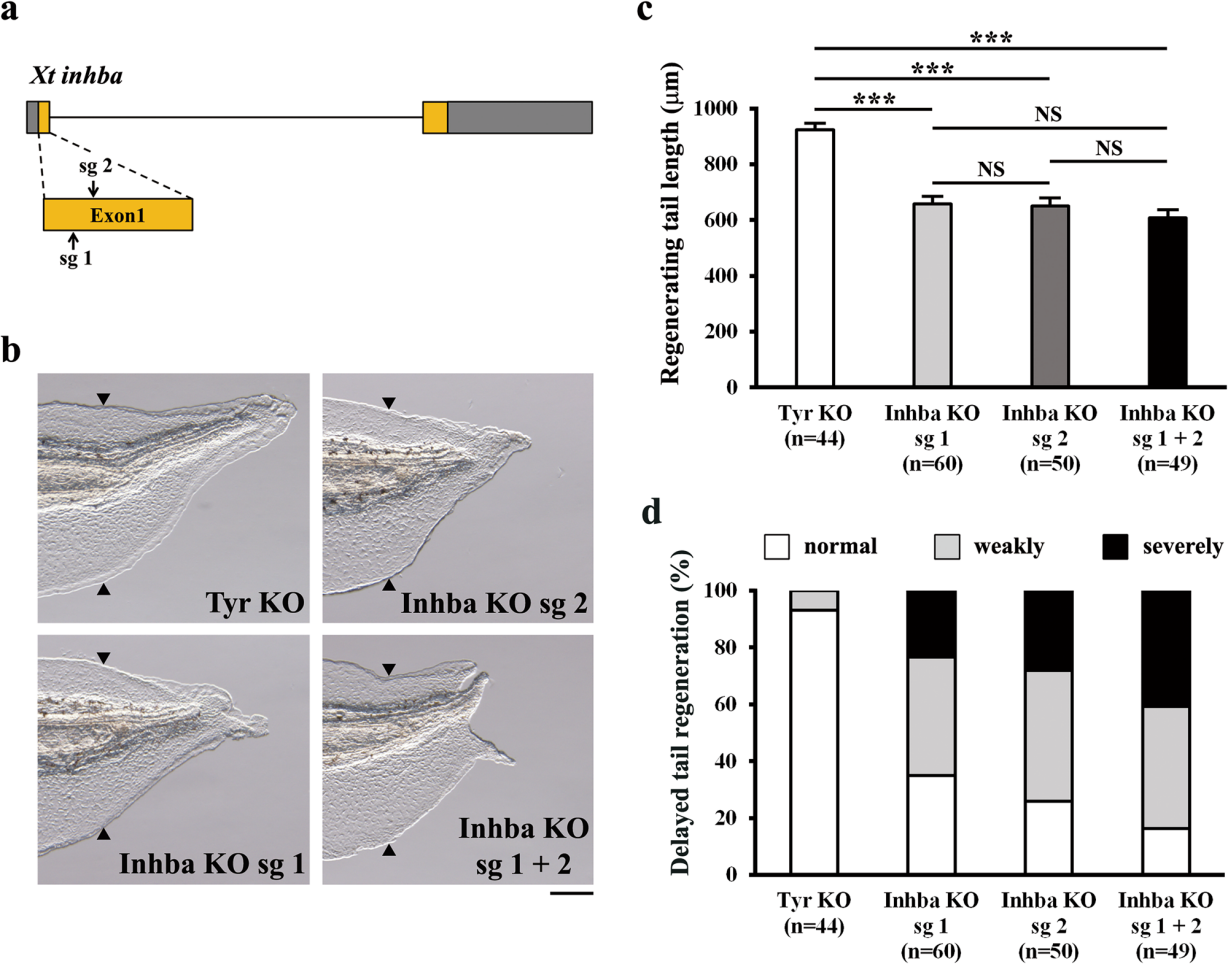
InhibinβA is required for tail regeneration. (**a**) Schematic drawing of sgRNA target sites (sg 1 and sg 2) in the *inhba* locus. Grey boxes, untranslated regions; orange boxes, coding regions; arrows, single-guide RNA target sites; bar, intron region. (**b**) Representative phenotypes of *tyr* KO (control), *inhba* KO sg 1, *inhba* KO sg 2, and *inhba* KO sg 1 + sg 2 tadpoles at 72 hpa. (**c**) The lengths of regenerating tails in KO tadpoles at 72 hpa. (**d**) Summary of phenotypes in KO tadpoles at 72 hpa. On the basis of the lengths of regenerating tails at 72 hpa, tadpoles were classified into three phenotypic groups (normal regeneration, weakly delayed regeneration, or severely delayed regeneration). Black arrowheads indicate amputation sites. Scale bar, 200 μm. NS, not significant; ***P < 0.001.

Since *inhba* was expressed in response to injury-activated signaling during tail regeneration, we investigated whether InhibinβA is essential for tail regeneration. We generated F0 *inhba* KO tadpoles using the CRISPR/Cas9 system: two different sgRNAs (sg 1 and sg 2) were designed using the genomic sequence corresponding to the pro-domain of InhibinβA; each sgRNA was injected into fertilized eggs (Fig. 3a). Both sgRNAs induced mutations in the *inhba* locus at moderate frequencies as determined by a T7 endonuclease I (T7E1) assay (Supplementary Figure 5). At 72 hpa, *inhba* KO tadpoles injected with each sgRNA showed a delay in tail regeneration, and the lengths of regenerating tails in *inhba* KO tadpoles were reduced compared to control tadpoles (*tyrosinase* (*tyr*) KO) (Fig. 3b and c). In addition, when classified according to the lengths of regenerating tails, a high proportion of *inhba* KO tadpoles showed delayed tail regeneration phenotypes (sg 1, 64.9%; sg 2, 74.0%) (Fig. 3d). The similarity of phenotypes of tadpoles injected with either sg 1 or sg 2 suggests that the retardation of tail regeneration in *inhba* KO tadpoles resulted from the specific inhibition of InhibinβA function. To increase the mutation efficiency, we injected a combination of sg 1 and sg 2 into fertilized eggs and found that 91.6% of alleles contained out-of-frame mutations with stop codons around the sgRNA target sites; 6.7% of alleles had in-frame mutations. In total, 98.3% of alleles had mutations in the *inhba* locus (Supplementary Figure 6). Moreover, the combination of sgRNAs increased the proportion of tadpoles displaying delayed tail regeneration (sg 1 + sg 2, 83.6%) compared with either sg 1 or sg 2 alone (Fig. 3d). Therefore, we used the combination of sg 1 and sg 2 in subsequent experiments. The delay in tail regeneration in *inhba* KO and *junb* KO tadpoles was specific as there was no significant delay in developing tadpole tails before tail amputation at stage 41/42; the delay in tail regeneration was observed even at 10 days post-amputation (dpa) (Supplementary Figure 7). In addition, the survival rate of these KO tadpoles was similar to that of the *tyr* KO control (Supplementary Figure 8). Finally, to further confirm the specificity of the phenotype observed in *inhba* KO, a rescue experiment was performed by overexpressing InhibinβA (Supplementary Figures 9 and 10). Overexpression of InhibinβA in *inhba* KO tadpoles partially but significantly rescued the shortened regenerating tail length and markedly reduced the proportion of tadpoles showing delayed tail regeneration. Taken together, we conclude that InhibinβA is required for regenerative outgrowth of the *Xenopus* tadpole tail.

**Figure 4:**
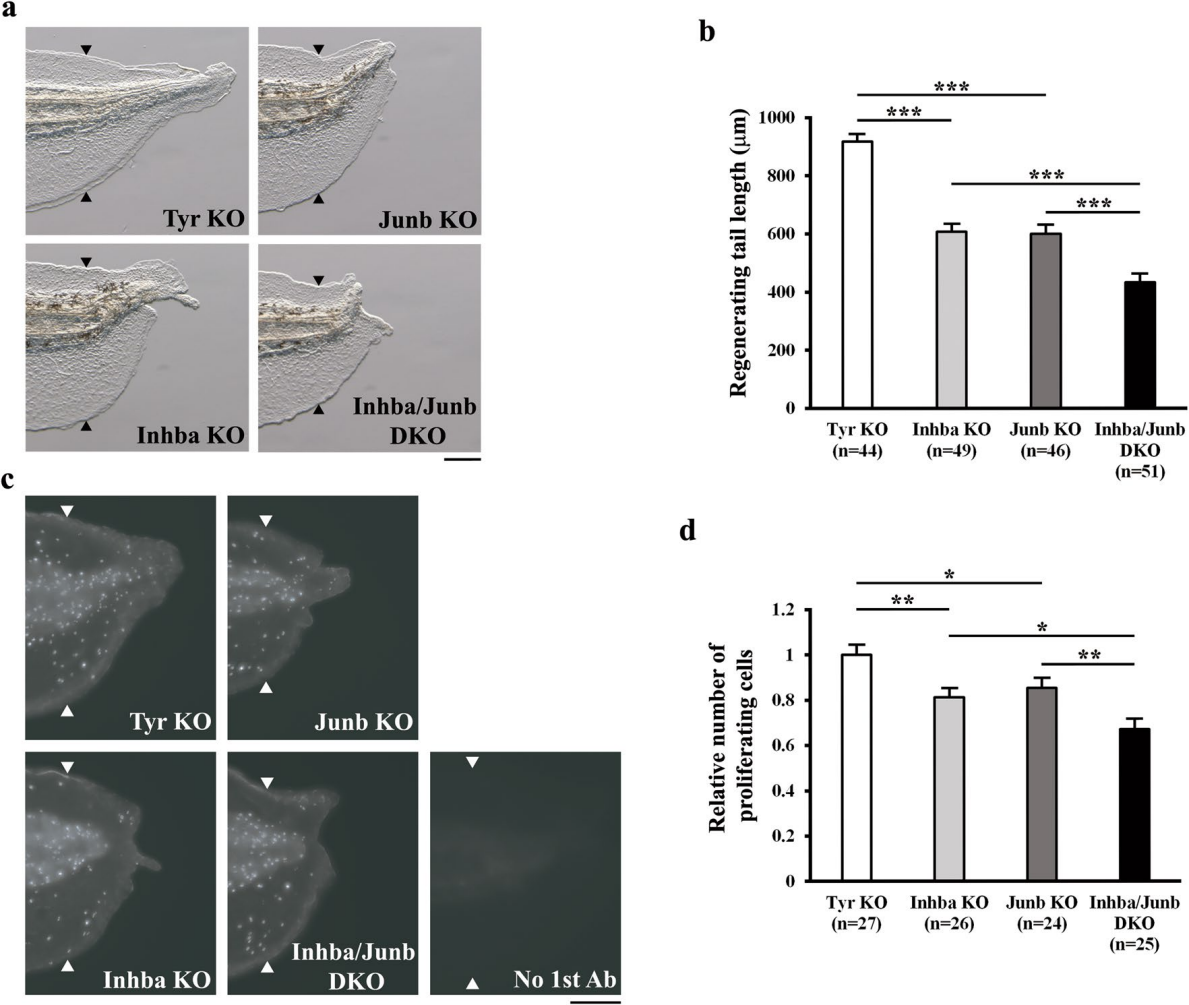
InhibinβA and JunB cooperate for regenerative outgrowth/cell proliferation. (**a**) Representative phenotypes of *tyr* KO (control), *inhba* KO, *junb* KO, and *inhba*/*junb* DKO tadpoles at 72 hpa. (**b**) The lengths of regenerating tails in KO and DKO tadpoles at 72 hpa. (**c**) Representative immunofluorescent images of pH3 staining (white dots) in KO and DKO tadpoles at 36 hpa. Whole-mount immunostaining was performed with the pH3 antibody; immunostaining without the pH3 antibody was used as a negative control (no 1st Ab). (**d**) Relative number of proliferating cells at 36 hpa. The number of pH3-positive cells was divided by the corresponding area. All values were normalized against the value of *tyr* KO. Black and white arrowheads indicate amputation sites. Scale bars, 200 μm. *P < 0.05, **P < 0.01, ***P < 0.001.

**Figure 5:**
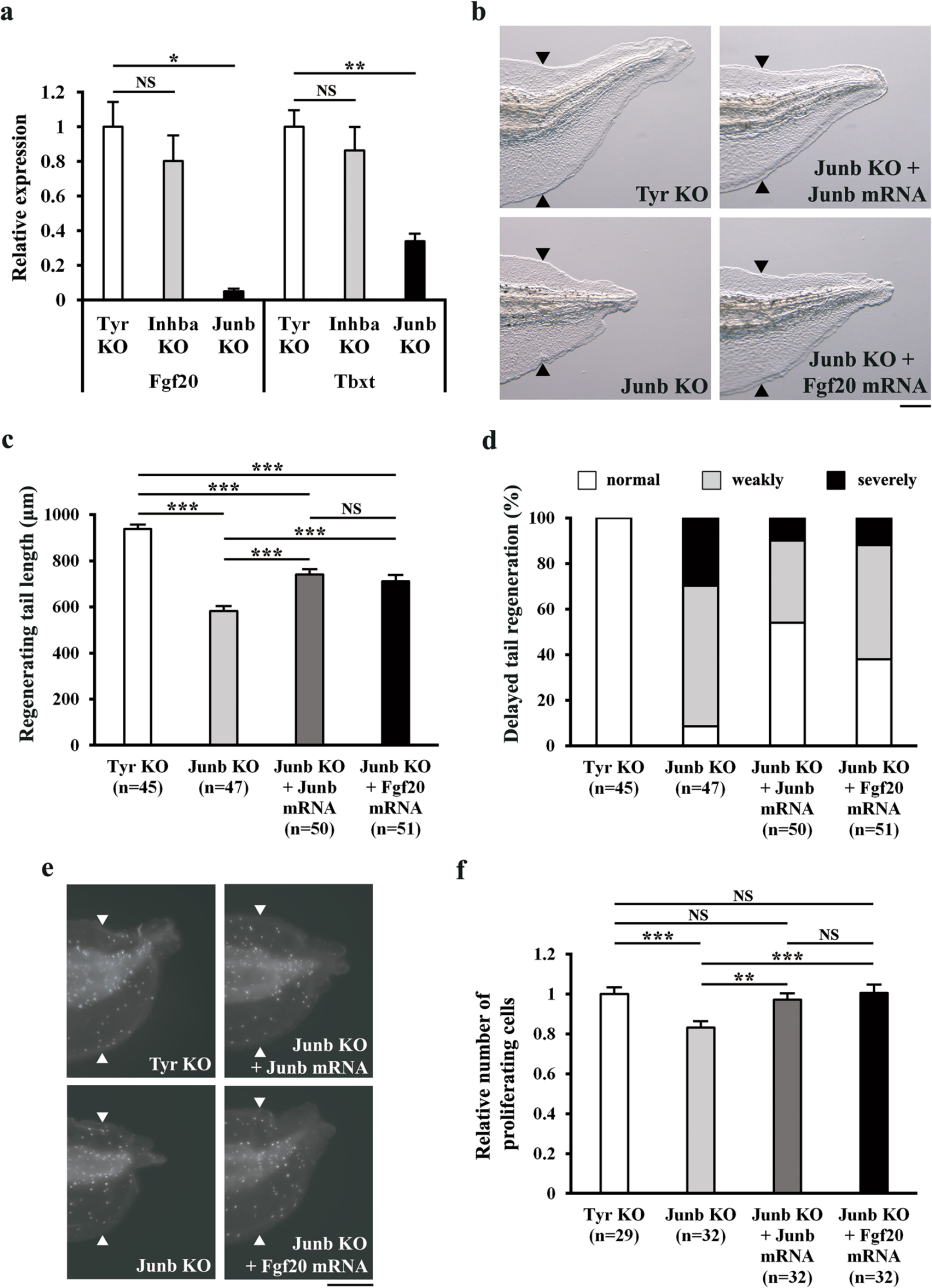
JunB regulates regenerative outgrowth/cell proliferation via FGF signaling. (**a**) Relative expression of *fgf20* and *tbxt* in *inhba* KO and *junb* KO tadpoles at 36 hpa. The data were normalized against the expression of *rps18*, and then by the value of *tyr* KO. (**b**) Representative phenotypes of *tyr* KO (control), *junb* KO, *junb* KO + *junb* mRNA, and *junb* KO + *fgf20* mRNA tadpoles at 72 hpa. (**c**) The lengths of regenerating tails in KO tadpoles at 72 hpa. (**d**) Summary of phenotypes in KO tadpoles at 72 hpa. On the basis of the lengths of regenerating tails at 72 hpa, tadpoles were classified into three phenotypic groups (normal regeneration, weakly delayed regeneration, or severely delayed regeneration). (**e**) Representative immunofluorescent images of pH3 staining (white dots) in *tyr* KO (control), *junb* KO, *junb* KO + *junb* mRNA, and *junb* KO + *fgf20* mRNA tadpoles at 36 hpa. (**f**) Relative number of proliferating cells at 36 hpa. The number of pH3-positive cells was divided by the corresponding area. All values were normalized against the value of *tyr* KO. Black and white arrowheads indicate amputation sites. Scale bars, 200 μm. NS, not significant; *P < 0.05, **P < 0.01, ***P < 0.001.

Next, we examined whether InhibinβA and JunB cooperate for regenerative outgrowth/cell proliferation. Double KO (DKO) of *inhba* and *junb* was performed by combining two sets of sgRNAs (two each for *inhba* and *junb*). In the DKO tadpoles, the mutation efficiencies at *inhba* and *junb* loci were comparable to those observed in the respective single KO tadpoles based on the T7E1 assay. As shown in Fig. 4a, b, the lengths of regenerating tails were shorter in *inhba*/*junb* DKO tadpoles than in *inhba* KO or *junb* KO tadpoles. Furthermore, whole-mount immunostaining of phosphorylated Histone H3 (pH3) in regenerating cells at 36 hpa revealed that the number of proliferating cells was significantly lower in *inhba*/*junb* DKO tadpoles than in *inhba* KO or *junb* KO tadpoles (Fig. 4c, d). Notably, there were no significant differences in the rates of cell proliferation in the proximal (headward) tail region of amputation sites among experimental groups (Supplementary Figure 11; see also Fig. 5e, f described below), suggesting that InhibinβA and JunB function in regenerating tissue to regulate regenerative cell proliferation. These findings provide strong evidence that InhibinβA and JunB cooperate for regenerative outgrowth/cell proliferation in the *Xenopus* tadpole tail.

### JunB regulates regenerative outgrowth/cell proliferation through FGF signaling

To elucidate the molecular mechanisms by which InhibinβA and JunB initiate regeneration processes, we searched for candidate downstream factors of InhibinβA and JunB during *Xenopus* tail regeneration. Inhibition of FGF signaling is known to affect *Xenopus* tail regeneration^9^. The FGF ligand *fgf20* is highly upregulated after wound healing, and morpholino-mediated knockdown of Fgf20 causes a delay in tail regeneration^18^. Moreover, regenerative outgrowth/cell proliferation of the zebrafish caudal fin is inhibited in *fgf20a* mutants^19^. We therefore examined whether InhibinβA and JunB regulate tail regeneration through FGF signaling. First, expression of *fgf20* and *tbxt* (also known as *brachyury*), a downstream effector of FGF signaling, were analyzed in regenerating cells of *inhba* KO and *junb* KO tadpoles at 36 hpa. A quantitative reverse transcription-PCR (qRT-PCR) analysis showed that *fgf20* and *tbxt* transcripts were downregulated to a significant extent in *junb* KO tadpoles (Fig. 5a), suggesting that JunB, but not InhibinβA, regulates the expression of *fgf20* and *tbxt* at the time when regenerating cells were actively proliferating. A rescue experiment was performed to determine whether JunB facilitates tail regeneration through FGF signaling. We overexpressed Fgf20 or JunB in *junb* KO tadpoles and measured the lengths of regenerating tails in the tadpoles. Our results indicated that Fgf20 partially but significantly rescued the delay in tail regeneration of *junb* KO tadpoles to the same extent as JunB (Fig. 5b, c; Supplementary Figure 10). Moreover, when classified according to the lengths of regenerating tails, the proportion of *junb* KO tadpoles with severely delayed tail regeneration was greatly reduced by Fgf20 overexpression (Fig. 5d). Next, we analyzed cell proliferation in these tadpoles at 36 hpa. As shown in Fig. 5e, f, the reduction in proliferating cells in *junb* KO tadpoles was rescued by overexpression of Fgf20 or JunB. Together with the finding of downregulated expression of *fgf20* and *tbxt* in *junb* KO tadpoles (described above), these data support the conclusion that JunB acts, at least in part, through FGF signaling to promote regenerative outgrowth/cell proliferation of the *Xenopus* tadpole tail.

### FGF signaling in the proliferation phase is important for regenerative outgrowth/cell proliferation

**Figure 6:**
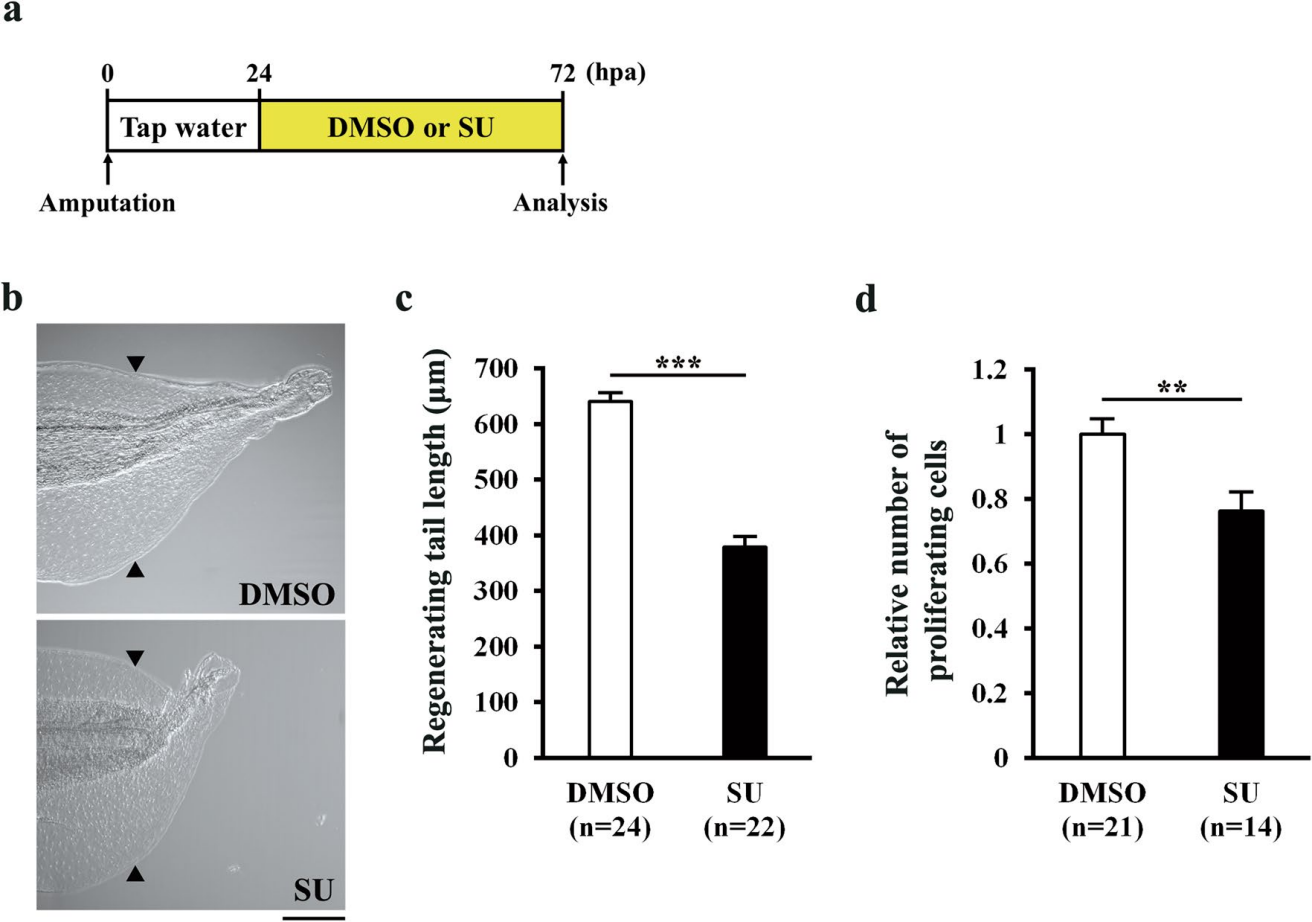
FGF signaling is required for regenerative outgrowth/cell proliferation. (**a**) Schematic of the experimental plan. Tadpoles were incubated in water containing 0.0375% DMSO (control) or 15 μM SU5402 (SU) from 24 hpa and analyzed at 72 hpa for regenerating tail length and proliferating cells. (**b**) Representative phenotypes of DMSO and SU-treated tadpoles at 72 hpa. (**c**) The lengths of regenerating tails at 72 hpa. (**d**) Relative number of proliferating cells at 72 hpa. The number of pH3-positive cells was divided by the corresponding area. In (**b**) and (**c**), the photograph and length of regenerating tails were obtained after whole-mount immunostaining, which involves bleaching and methanol treatments. Due to these treatments, the measured regenerating tail length is relatively shorter than in other experiments. All values were normalized against the value of DMSO-treated tadpoles. Black arrowheads indicate amputation sites. Scale bar, 200 μm. **P < 0.01, ***P < 0.001.

**Figure 7:**
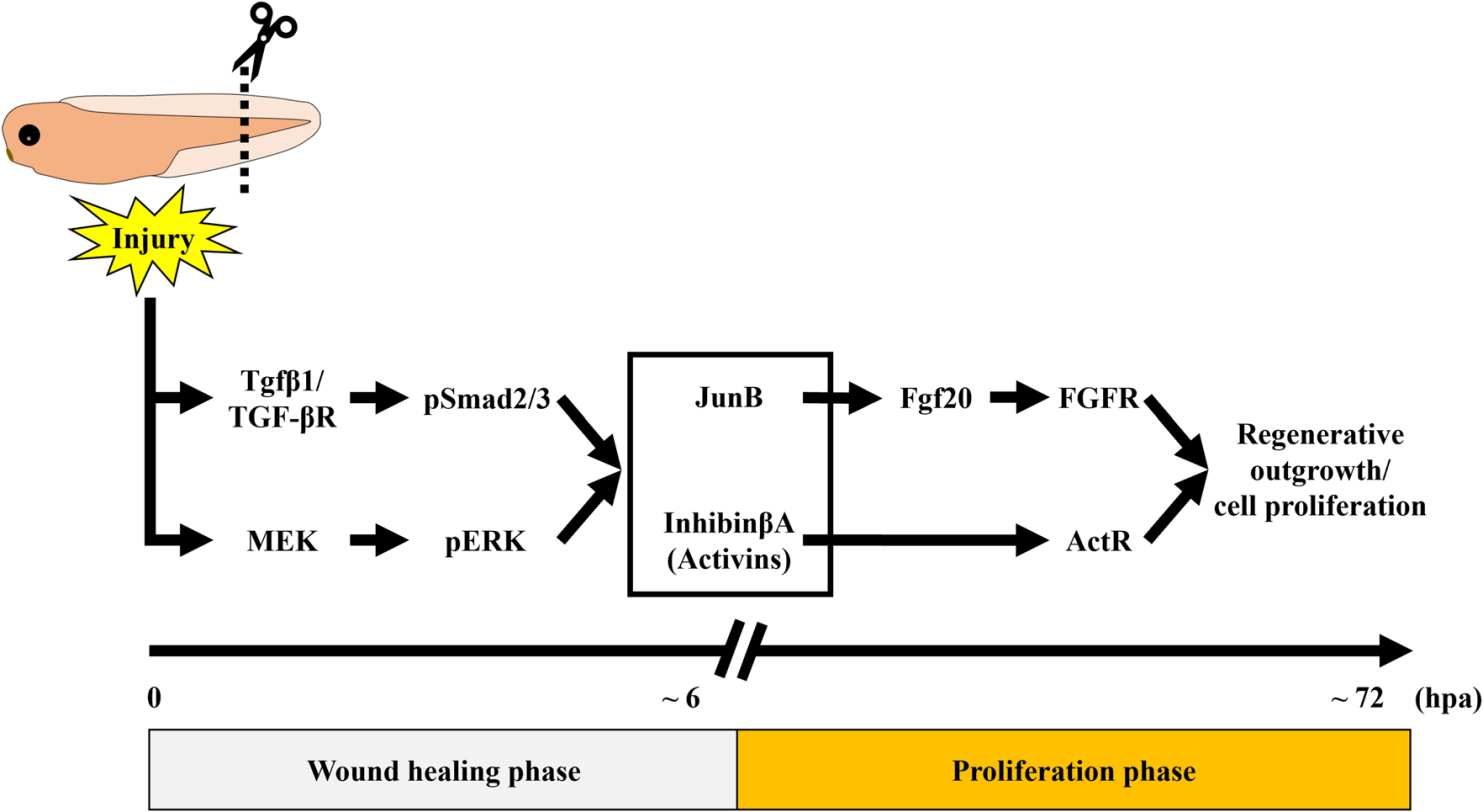
A model for the transition from wound healing to regenerative cell proliferation mediated by InhibinβA and JunB. Upon tail amputation, Tgfβ1/TGF-β receptor (TGF-βR) and MEK independently phosphorylate/activate Smad2/3 (pSmad2/3) and ERK (pERK), respectively. These injury-activated signals coordinately induce the expression of *inhba* and *junb* during the wound healing phase. After wound healing, InhibinβA and JunB cooperate to initiate outgrowth and cell proliferation. Mechanistically, JunB promotes tail regeneration, at least in part, via Fgf20/FGF receptor (FGFR) signaling; InhibinβA/Activin receptor (ActR) signaling may act either in parallel with or downstream of Fgf20/FGFR signaling (see Discussion).

FGF signaling is well known to be essential for tissue/organ regeneration in regenerative animals^9,12,14,18–20^. Although inhibition of FGF signaling causes a delay in *Xenopus* tail regeneration^9^, the role of FGF signaling in regenerative cell proliferation is still unclear. In addition, the expression of *fgf20*, which functions downstream of injury-induced JunB, increases as outgrowth/cell proliferation progresses in the regenerating *Xenopus* tail^18^, and inhibition of FGF signaling does not affect wound healing^9^. Therefore, we speculated that FGF signaling in the proliferation phase might be required for outgrowth/cell proliferation in *Xenopus* tail regeneration. To suppress FGF signaling specifically in the proliferation phase, tail-amputated tadpoles were treated with SU5402 (SU, FGF receptor inhibitor) from 24 hpa (the time when regenerative outgrowth begins) and the lengths of regenerating tails were measured at 72 hpa (Fig. 6a). Consistent with a previous report^9^, inhibition of FGF signaling in the proliferation phase caused a delay in tail regeneration compared with DMSO-treated tadpoles (Fig. 6b, c). We then analyzed cell proliferation in SU-treated tadpoles and found that the number of proliferating cells in the regenerating tail was considerably reduced by SU (Fig. 6d). Thus, our results demonstrate that FGF signaling in the proliferation phase is essential for regenerative outgrowth/cell proliferation of the *Xenopus* tadpole tail. Given the fact that JunB, but not InhibinβA, regulates tail regeneration via Fgf20 (Fig. 5), we conclude that FGF signaling downstream of JunB functions in parallel with InhibinβA signaling in the proliferation phase (Fig. 7; see Discussion).

## Discussion

Injury-induced cell proliferation is a shared feature in animal species that have the ability to regenerate tissues. However, it is still not clear what factors are induced in response to injury to promote cell proliferation during tissue regeneration. Here, we show that *inhba* and *junb* are induced through injury-activated TGF-β/Smad and MEK/ERK signaling during the wound healing phase. Moreover, our study suggests the cooperative function of InhibinβA and JunB in injury-induced cell proliferation and tail regeneration. We propose that InhibinβA and JunB are important factors that mediate the transition from wound healing to regenerative cell proliferation during *Xenopus* tail regeneration (Fig. 7). The significance and possible mechanisms of the cooperative regulation of cell proliferation by InhibinβA and JunB are discussed in detail below.

Upon injury, multiple cell signaling pathways (TGF-β/Smad, ERK, and ROS) are known to be activated in the wound healing phase of the regenerating *Xenopus* tail^4,8,18^. However, the molecular mechanisms by which these injury-activated signals control regenerative outgrowth/cell proliferation through the regulation of gene expression are not well understood. We found that at the beginning of wound healing (~ 2 hpa), the phosphorylation of Smad2/3 was not dependent on MEK/ERK signaling (Supplementary Figure 3). Conversely, a previous report showed that the phosphorylation of ERK is not affected by inhibition of TGF-β/Smad signaling^8^. These observations imply that TGF-β/Smad and MEK/ERK signaling are independently activated by injury and coordinately regulate the expression of *inhba* and *junb* during wound healing (Figs. 1 and 7). While the upstream factors that activate MEK/ERK have not been elucidated, Tgfβ1 is required for the injury-induced phosphorylation of Smad2/3 (Supplementary Figure 3)^17^. Interestingly, *tgfb1* is already highly expressed in the uncut *Xenopus* tail^4,17^; after tail amputation, Tgfβ1 stored in the extracellular matrix is expected to induce expression of downstream target genes, including *inhba* and *junb*, via the activation of Smad2/3. We postulate that MEK/ERK signaling may help to refine the expression of *inhba* and *junb* induced by the Tgfβ1-Smad2/3 pathway. The injury-induced burst of *inhba* and *junb* expression is fast and takes place within a few hours (~ 6 hpa). Therefore, it is possible that the coordination of TGF-β/Smad and MEK/ERK signaling is essential for a sharp and sustained increase in *inhba* and *junb* expression after tail amputation, ultimately contributing to the successful recovery from injury and the regeneration of damaged tissue*.*

The induction of *inhba* expression by the Tgfβ1-Smad2/3 pathway indicates the amplification of TGF-β/Smad signaling during *Xenopus* tail regeneration (Fig. 7). This is consistent with the observation that expression of TGF-β family ligands (*inhba* and *gdf11*) is sequentially induced during tail regeneration^4^. As described above, the activation of Smad2/3 is not suppressed by inhibition of MEK/ERK signaling at the beginning of wound healing. In contrast, at the end of wound healing, the phosphorylation of Smad2/3 is reduced by MEK/ERK inhibitor^8^. We speculate that the reduction of activated Smad2/3 at the late time point of wound healing may be due to the disruption of the amplification system of TGF-β/Smad signaling by downregulation of *inhba* after MEK/ERK inhibition (Fig. 1a). Furthermore, the core components of TGF-β signaling (ligands, receptors, and their transcriptional target genes) were found to be co-expressed during axolotl limb regeneration^21^. Given that TGF-β signaling plays critical roles in multiple steps of regeneration processes^4,22–24^, it is tempting to suggest that the amplification of TGF-β/Smad signaling is conserved among animal species with a high regenerative capacity and is fundamental for maintaining TGF-β signaling throughout regeneration.

Unlike urodeles, regenerating axial tissues (spinal cord, notochord, and somite) in the *Xenopus* tail have been shown to originate from the respective differentiated tissues in the tail stump, and metaplasia of tissue progenitors is not observed^25^. In addition, dedifferentiation phenotypes are not found and regenerating myofibers are derived from skeletal muscle tissue stem cells (satellite cells). These observations indicate that tissue renewal mediated by the induction of cell proliferation in differentiated cells is a critical step of *Xenopus* tail regeneration. Therefore, while InhibinβA and JunB could regulate differentiation directly or indirectly at some stages of regeneration, it is plausible that the cooperative function of InhibinβA and JunB in regenerative cell proliferation is important for successful tissue recovery. It will be interesting to analyze the contribution of this cooperation in other animal species with different regenerative capacities and cellular plasticities, particularly in urodeles.

During spinal cord regeneration in the axolotl, cell proliferation is accelerated by the progression of the cell cycle from the G0/G1 to S phase^26^, indicating that the release from the G0/G1 phase is a crucial step in regenerative cell proliferation. Notably, it has been shown that inhibition of InhibinβA or JunB in cultured cell lines increases the proportion of cells in the G0/G1 phase^27,28^. Moreover, in the present study, impairment of regenerative cell proliferation in *inhba*/*junb* DKO was greater than in either *inhba* KO or *junb* KO (Fig. 4d). Therefore, in response to tail amputation, InhibinβA and JunB may work together in *Xenopus* to promote cell cycle re-entry by facilitating the release from the G0/G1 phase. It has been reported that both Smad2/3 (signal transducers of InhibinβA) and JunB are essential for the transcription of cell cycle-related genes^29,30^ and that Smad3 regulates target gene expression by binding to JunB^31–34^. These findings and our results suggest that the cooperative function of InhibinβA and JunB in regenerative cell proliferation might be mediated by a transcriptional complex composed of Smad2/3 and JunB during *Xenopus* tail regeneration. In this case, InhibinβA-activated Smad2/3 and JunB directly converge in the cell cycle regulatory system. In parallel with this potential mechanism, InhibinβA may also interact with downstream effectors of JunB in the regulation of cell proliferation. Intriguingly, we found that Fgf20 acted downstream of JunB in *Xenopus* tail regeneration (Fig. 5). In addition, Activin A, a homodimer of InhibinβA, requires FGF signaling in mesoderm formation during *Xenopus* embryogenesis and in the endodermal differentiation of mouse embryogenic stem cells^35–37^. As the expression of *fgf20* and its target gene *tbxt* was not reduced in *inhba* KO tadpoles when regenerating cells were actively proliferating (Fig. 5a), it is possible that InhibinβA functions with FGF signaling downstream of *tbxt* expression to regulate tail regeneration. Tbxt is known to associate with Smad2/3, and the genomic binding sites of Tbxt overlap with those of Smad2/3 in differentiating human embryonic stem cells^38^. An alternative but not mutually exclusive possibility is that InhibinβA affects other signaling pathways involved in regeneration processes. During regeneration of the zebrafish fin and *Xenopus* tadpole tail, Activin/TGF-β signaling regulates the expression of the BMP target gene *msx1*^4,24^. Since BMP signaling is required for *Xenopus* tail regeneration^10^, InhibinβA might promote tail regeneration in cooperation with JunB by modulating the BMP pathway. Overall, the collaborative action of InhibinβA and JunB in regenerative outgrowth/cell proliferation may be mechanistically important for the robust patterning of regenerating tissue; this possibility will be investigated in future studies.

In conclusion, we showed that *inhba* and *junb* are coordinately induced upon tail amputation through TGF-β/Smad and MEK/ERK signaling, and that injury-induced InhibinβA and JunB cooperate to drive regenerative outgrowth/cell proliferation in the *Xenopus* tadpole tail. These findings provide valuable insights into the molecular mechanisms that mediate the transition from wound healing to regenerative cell proliferation during tissue regeneration.

## Methods

### Animals, microinjection, and regeneration assay

All experiments were conducted in accordance with ARRIVE guidelines and with the Fundamental Guidelines for Proper Conduct of Animal Experiment and Related Activities in Academic Research Institutions under the jurisdiction of the Ministry of Education, Culture, Sports, Science and Technology of Japan, and were approved by the Hiroshima University Animal Research Committee (Permit Number: G19-7-2). *X. tropicalis* embryos were cultured in 0.1X Marc’s Modified Ringer solution (MMR) at 26 °C. Tadpoles at stage 41/42 were anesthetized in 0.05% MS-222/0.01X MMR and subjected to tail amputation at the mid-point (50%) of tail length using a surgical knife. The removal of as much as 75% of tail length does not affect tail regeneration or the survival of tadpoles^39^. The amputated tadpoles were maintained until 72 hpa or 10 dpa in tap water. For inhibitor experiments, tadpoles were treated with 25 μM SB-505124 (Cayman Chemical) or 0.25 μM PD0325901 (Cayman Chemical) from 1 h before tail amputation, or with 15 μM SU5402 (Sigma) from 24 hpa.

### Cloning of *X. tropicalis inhba* and *fgf20*

Full-length cDNAs for *inhba* (GenBank Accession number XM_002933406.3) and *fgf20* (GenBank Accession number NM_001143927.1) were PCR-amplified using the following primers: *inhba*, forward 5′-CGG GAT CCC ACC TGG TGA CAG GAT GC-3′ and reverse 5′-GCT CTA GAA ATT GCT GCA GGC TGG TAA C-3′; *fgf20*, forward 5′-CGG GAT CCC TTT TGG GGA TTT TGG GAC T-3′ and reverse 5′-AAG GCC TGC ACT GGG TTT GGT TTG TCT-3′. The PCR products from *inhba* and *fgf20* were cloned into the BamHI/XbaI and BamHI/StuI sites of *pDH105* (*pDH105-inhba* and *pDH105-fgf20*), respectively.

### CRISPR/Cas9-mediated KO, rescue experiment, and genotyping

CRISPR/Cas9-mediated mutagenesis was carried out as previously described^15,40^. For the KO experiment, 1000 pg of sgRNA was used. In the DKO experiment, 500 pg each of the two different sgRNAs for *inhba* and *junb* were used; the total amount of sgRNAs was 2000 pg. The injection solution was prepared by mixing sgRNAs and 1 ng of Cas9 protein (Integrated DNA Technologies) per 1000 pg of sgRNAs. For the rescue experiment, the capped *junb, inhba*, and *fgf20* mRNAs were synthesized from *pDH105-junb*^41^, *pDH105*-*inhba*, and *pDH105-fgf20*, respectively, using the SP6 transcription kit (Invitrogen). mRNA was mixed with sgRNAs and Cas9 protein, and the mixture was injected into the fertilized eggs. The sequences of sgRNAs for *tyr*, *junb*, and *tgfb1* were described in previous studies^15,17,40^, and primers for generating *inhba* sgRNAs were as follows: sg 1, 5′-ATT TAG GTG ACA CTA TAG GCC CCA ACT CCA GGA TCT GGT TTT AGA GCT AGA AAT AGC AAG-3′; sg 2, 5′-ATT TAG GTG ACA CTA TAG GCC ATG TCA CTC TGA GAA CGT TTT AGA GCT AGA AAT AGC AAG-3′. Genomic DNA was extracted from *inhba* KO tadpoles at 72 hpa and purified by the GenElute mammalian genomic DNA miniprep kit (Sigma). PCR amplification of the genomic region of *inhba* was performed using Q5 High Fidelity DNA polymerase (New England Biolabs). The PCR products were treated with T4 polynucleotide kinase (New England Biolabs) and cloned into the pUC57 vector, which was digested by EcoRV and dephosphorylated with Quick CIP (New England Biolabs). To determine the mutation types, single colonies were subjected to Sanger sequencing. T7E1 (New England Biolabs) assay was performed as previously described^15^. The following primers were used for PCR and sequencing: *inhba*, forward 5′-ACA GCC ACA AAT ACC CAC AG-3′ and reverse 5′-AAG GAG CCA GTG AAG CTT TG-3′.

### RNA isolation and qRT-PCR

RNA isolation was performed as previously described^15^. In brief, regenerating tails were surgically isolated from 15 to 30 *Xenopus* tadpoles and lysed in a solution containing proteinase K. RNA was purified by phenol/chloroform extraction and ethanol precipitation. cDNA was synthesized using the PrimeScript RT reagent kit (Takara), and qRT-PCR was performed using THUNDERBIRD Next SYBR qPCR Mix (TOYOBO) and CFX Connect Real-Time System (BIO-RAD). Each qRT-PCR experiment was performed in triplicate. To detect endogenous mRNAs, the following primers were used for qRT-PCR: *inhba*, forward 5′-CAT GTG ATC AAT GCC AGG AG-3′ and reverse 5′-TCT TCC TCA ACA CCA GAT GC-3′; *fgf20*, forward 5′-TCG CAT CTC CAG GGA ATC-3′ and reverse 5′-GAT GAA TTC AAG GAT ACC AAA GC-3′; *tbxt*, forward 5′-CAA TTC CCC AAA CAA TTT AGC-3′ and reverse 5′-TGA CTT GAG GTA GGA GGT GTT C-3′; the primer sequences of *junb* and *rps18* were described in the previous study^15^. To detect injected mRNAs, the following primers were used: *inhba*, forward 5′-AAG TCA GAG TTA TCT GGG TTT GG-3′ and reverse 5′-GCA TTC TAG TTG TGG TTT GTC C-3′; *fgf20*, forward 5′-GCC AAA GAC AAA CCA AAC CCA-3′ and reverse 5′-TGG ATC TAC GTA ATA CGA CTC ACT-3′; *junb*, forward 5′-TTA CTG GGA AAG GGC ATC AG-3′ and reverse 5′-GCA TTC TAG TTG TGG TTT GTC C-3′.

### Whole-mount in situ hybridization and whole-mount immunostaining

Whole-mount in situ hybridization and cryosectioning of hybridized tadpoles were performed as previously described^42–44^. Tadpoles were fixed in MEMFA solution for 2 h at room temperature and stored in 100% ethanol at − 20 °C. For anti-sense and sense probes, *pDH105-inhba* was linearized by BamHI and XbaI and subjected to in vitro transcription by T3 and SP6 RNA polymerases (Roche), respectively. For the whole-mount immunostaining, tadpoles were fixed in MEMFA solution for 30 min at room temperature and stored in 100% methanol at − 20 °C. Whole-mount immunostaining was carried out following our standard protocol for pH3 staining^15^ and with a minor modification for pSmad2/3 staining^17^. The following antibodies were used at 1:500 dilution: anti-pH3 antibody (Upstate Biotechnology), anti-pSmad2/3 antibody (Cell Signaling Technology), Alexa Fluor 488 goat anti-rabbit antibody (Molecular Probes, Thermo Fisher Scientific), and Alexa Fluor 488 goat anti-mouse antibody (Molecular Probes, Thermo Fisher Scientific).

### Microscopy, quantification, and statistical analysis

Fluorescent images were obtained with Axio Zoom.V16 (ZEISS), and the fluorescence intensity was quantified with ZEN blue software (ZEISS). Images of cryosections were captured using an Axio Observer.Z1 (ZEISS). Quantification of pSmad2/3 staining was performed as described previously^17^. The length of the regenerating tail was measured with cellSens Standard imaging software (Olympus) or ImageJ software (National Institutes of Health, USA). On the basis of the lengths of regenerating tails at 72 hpa, tadpoles were classified as showing normal regeneration (more than 80% of the average length of *tyr* KO), weakly delayed regeneration (60–80% of the average length of *tyr* KO), or severely delayed regeneration (less than 60% of the average length of *tyr* KO). The number of pH3-positive cells was manually counted in the regenerating tail, excluding the fin. Statistical analysis was performed by Student’s *t*-test with or without Bonferroni correction, and error bars indicate the standard error of the mean.

### Supplementary Information

Supplementary Information.

## Data Availability

No dataset was generated in this study. The GenBank accession numbers of *inhba* and *fgf20* genes isolated in this study are given in the Methods.
