# Supplementary material for: Injury-induced cooperation of InhibinβA and JunB is essential for cell proliferation in Xenopus tadpole tail regeneration
Source: Sci Rep. 2024 Feb 14;14:3679. doi: 10.1038/s41598-024-54280-w (PMC10867027; doi:10.1038/s41598-024-54280-w)

Injury-induced cooperation of InhibinβA and JunB is essential for cell proliferation in *Xenopus* tadpole tail regeneration

Makoto Nakamura, Tatsuya Kyoda, Hitoshi Yoshida, Kimiko Takebayashi-Suzuki, Ryota Koike, Eri Takahashi, Yuka Moriyama, Marcin Wlizla, Marko E. Horb and Atsushi Suzuki

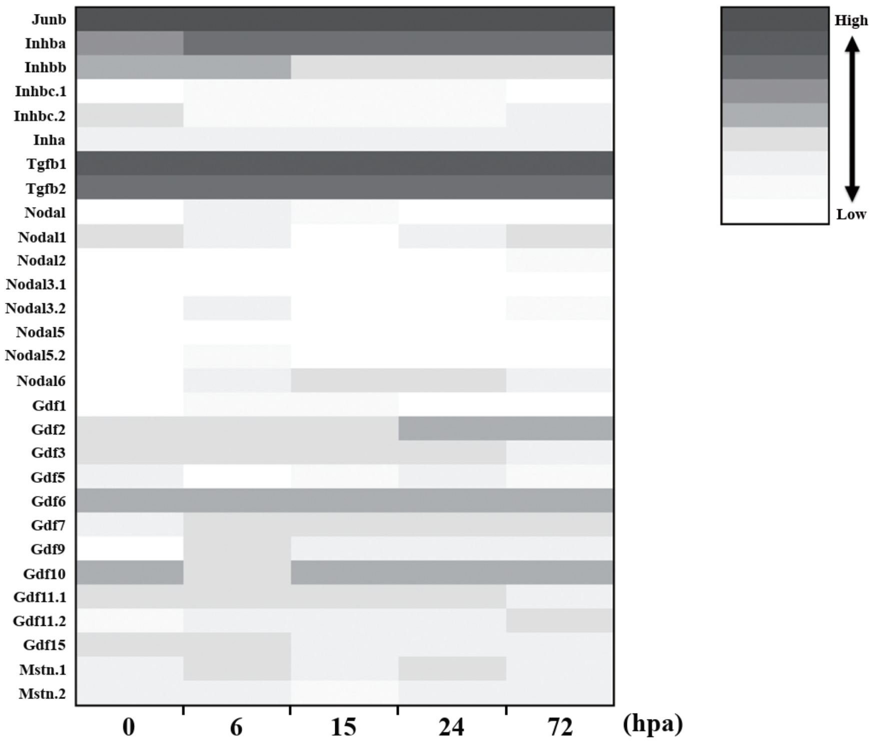

Supplementary Figure 1. High expression levels of *inhba*, *tgfb1*, and *tgfb2* compared to other TGF-β/Activin family ligands during tail regeneration

Heat map showing the expression levels of TGF-β/Activin family ligands and *junb* on a log scale of RNA-seq counts. hpa, hours post-amputation.

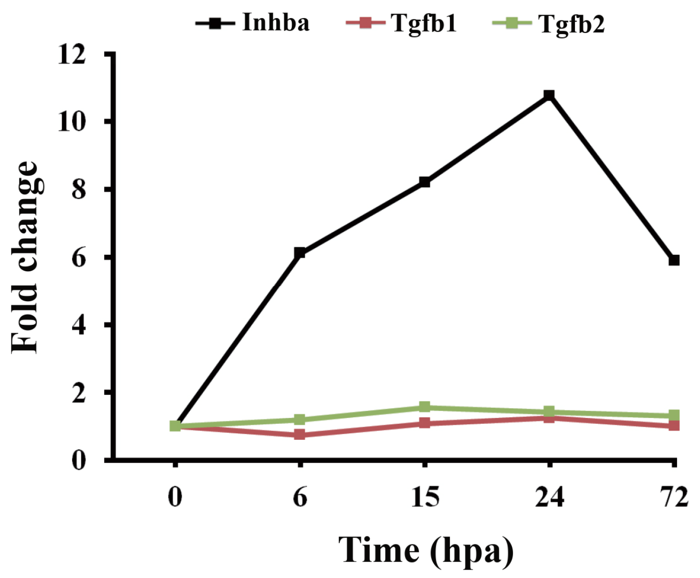

Supplementary Figure 2. Upregulation of *inhba* during tail regeneration

Fold changes in expression (relative to 0 hpa = 1) of *inhba*, *tgfb1*, and *tgfb2* at 0, 6, 15, 24, and 72 hpa.

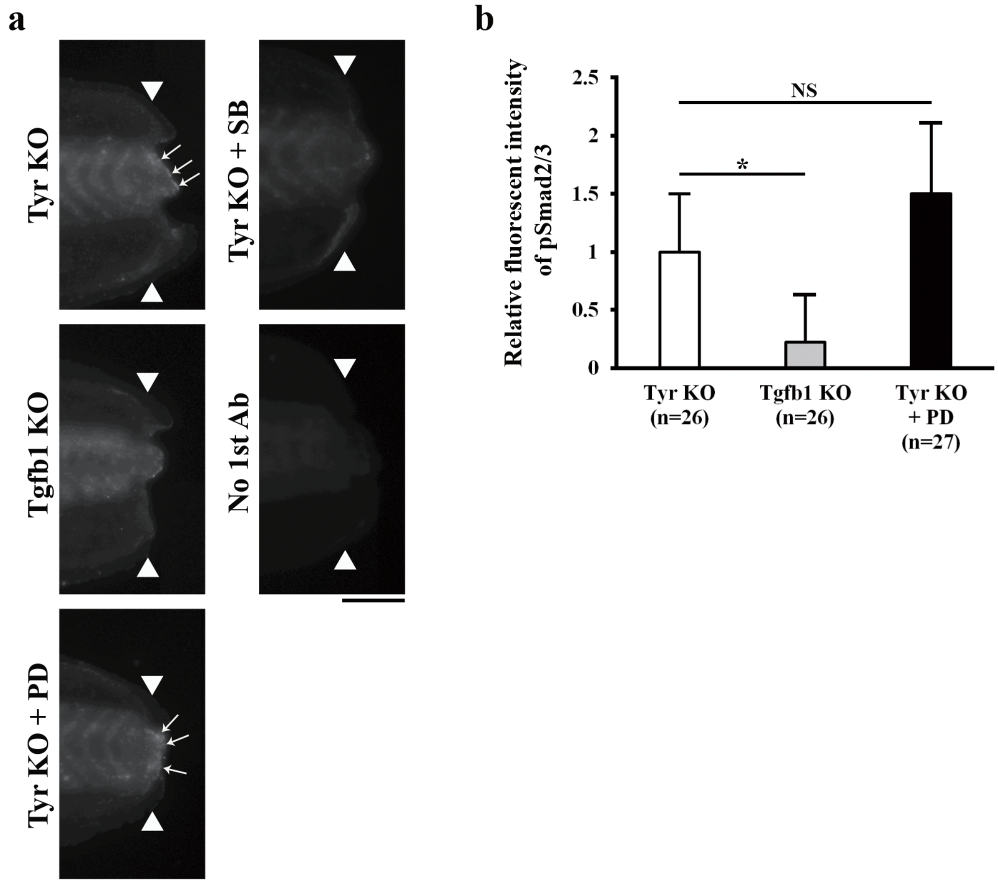

Supplementary Figure 3. Activation of Smad2/3 is not dependent on MEK/ERK signaling at the beginning of wound healing

(**a**) Representative immunofluorescent images of pSmad2/3 staining in *tyr* KO (control), *tgfb1* KO, 0.25 μM PD0325901 (PD)-treated *tyr* KO, and 25 μM SB-505124 (SB)-treated *tyr* KO tadpoles at 2 hpa. Whole-mount immunostaining was performed with the pSmad2/3 antibody; immunostaining without pSmad2/3 antibody was used as a negative control (no 1st Ab). (**b**) Quantitative analysis of pSmad2/3 fluorescent intensity in the regenerating tail. To detect TGF-β signal-dependent Smad2/3 phosphorylation, the mean value measured in SB-treated *tyr* KO was subtracted from that obtained in *tyr* KO, *tgfb1* KO, and PD-treated *tyr* KO. All values were normalized against the value of *tyr* KO. White arrows and arrowheads indicate pSmad2/3 staining and amputation sites, respectively. Scale bar, 200 μm. NS, not significant; *$P < 0.05$.

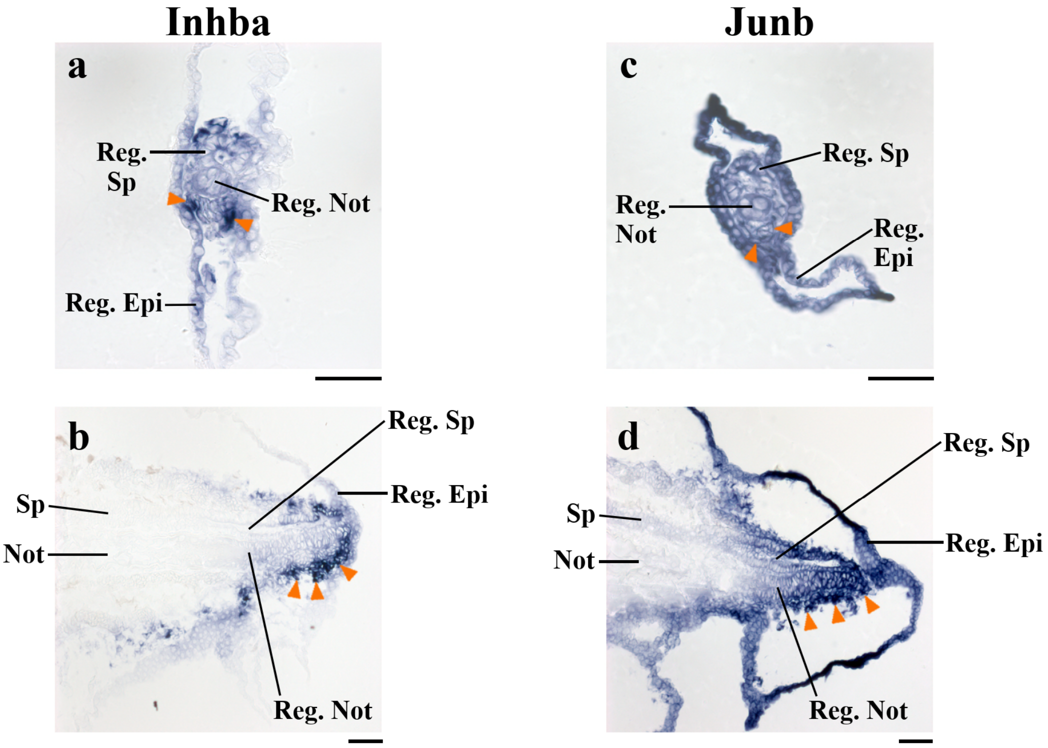

Supplementary Figure 4. Sections of tadpoles after whole-mount *in situ* hybridization for *inhba* and *junb*

Transverse (**a**, **c**) and sagittal (**b**, **d**) sections (10 μm) of regenerating tails at 48 hpa. Not, notochord; Sp, spinal cord; Reg. Not, regenerating notochord; Reg. Sp, regenerating spinal cord; Reg. Epi, regenerating epidermis. Orange arrowheads indicate regenerating mesenchyme. Scale bars, 50 μm.

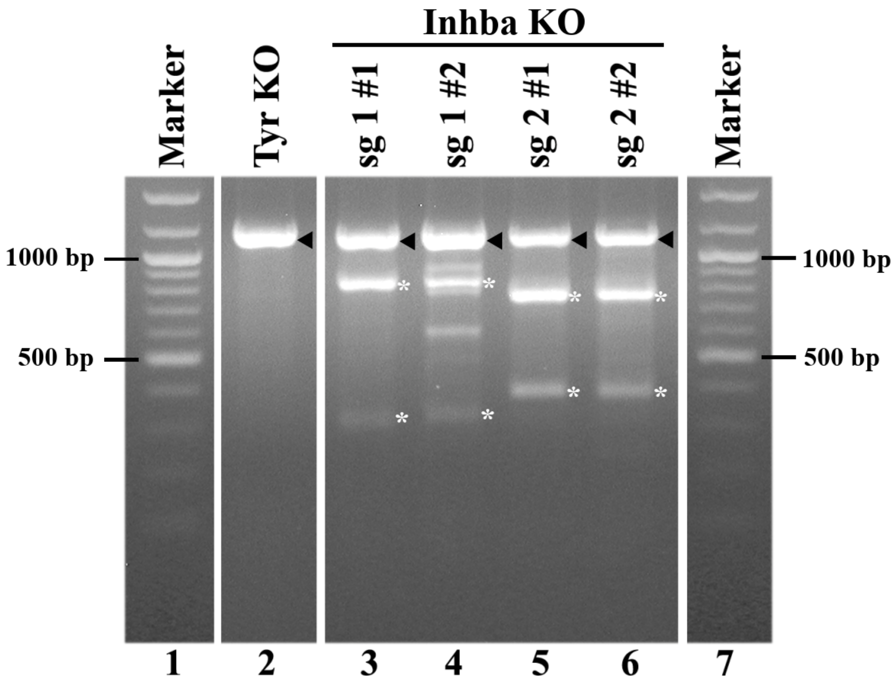

Supplementary Figure 5. Mutation analysis of *inhba* KO tadpoles using T7E1 assay

Tadpoles injected with *inhba* sg 1 and sg 2 were individually collected at 72 hpa. Two tadpoles (#1 and #2) were analyzed for each sgRNA. *tyr* KO is shown as a control. Black arrowheads and white asterisks indicate undigested and expected digested bands, respectively.

a

b

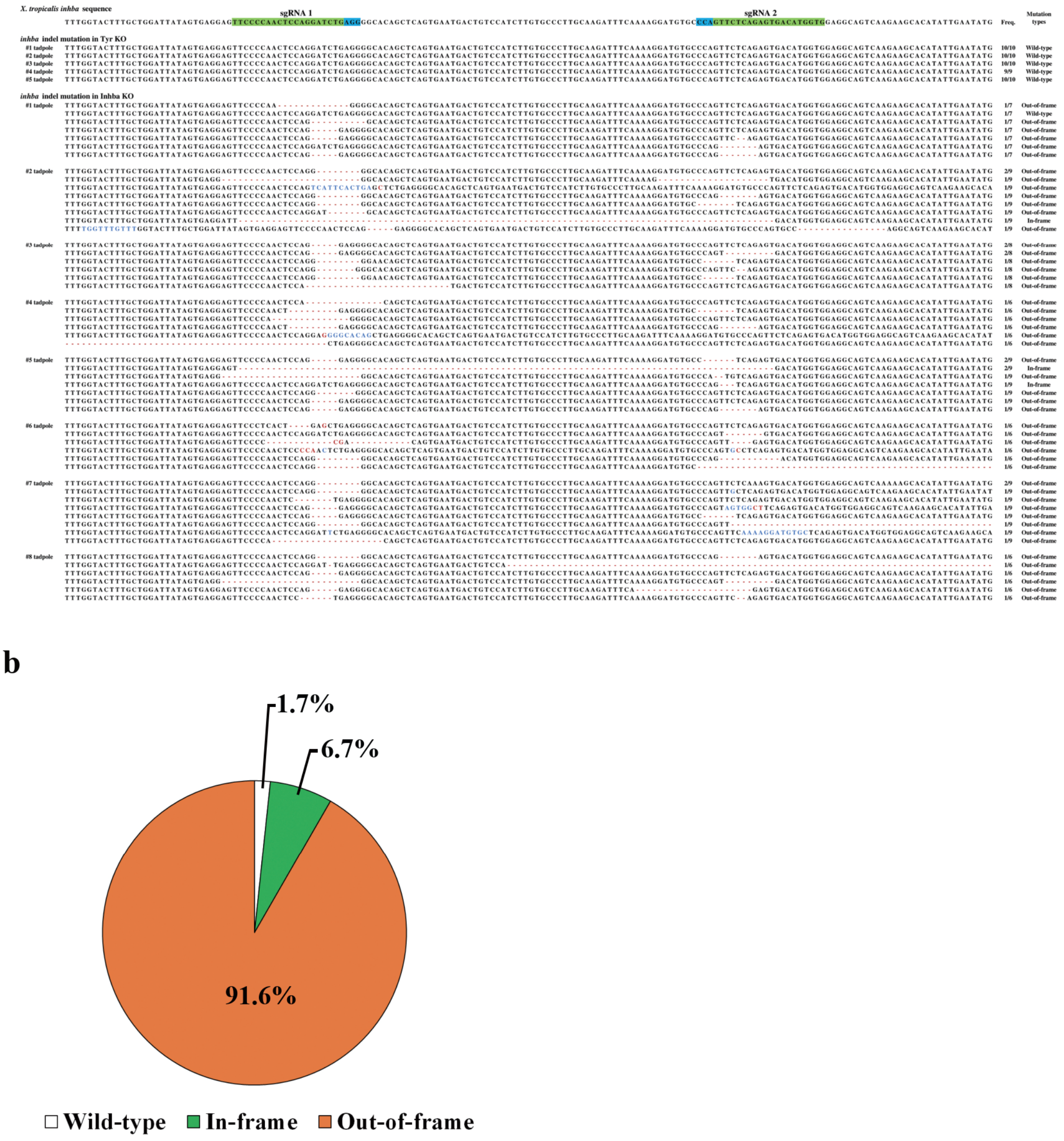

Supplementary Figure 6. Genotyping of *inhba* KO tadpoles

(**a**) Sequence analysis of *inhba* mutations in *tyr* KO (n = 5) and *inhba* KO sg 1 + sg 2 (n = 8) tadpoles. sgRNA target sites and PAM sequences are highlighted in green and blue, respectively. Red dashes indicate the deleted sequences, and red and blue letters show substitutions and insertions, respectively. (**b**) The proportion of mutation types (wild-type, in-frame, and out-of-frame) in *inhba* KO sg 1 + sg 2 tadpoles.

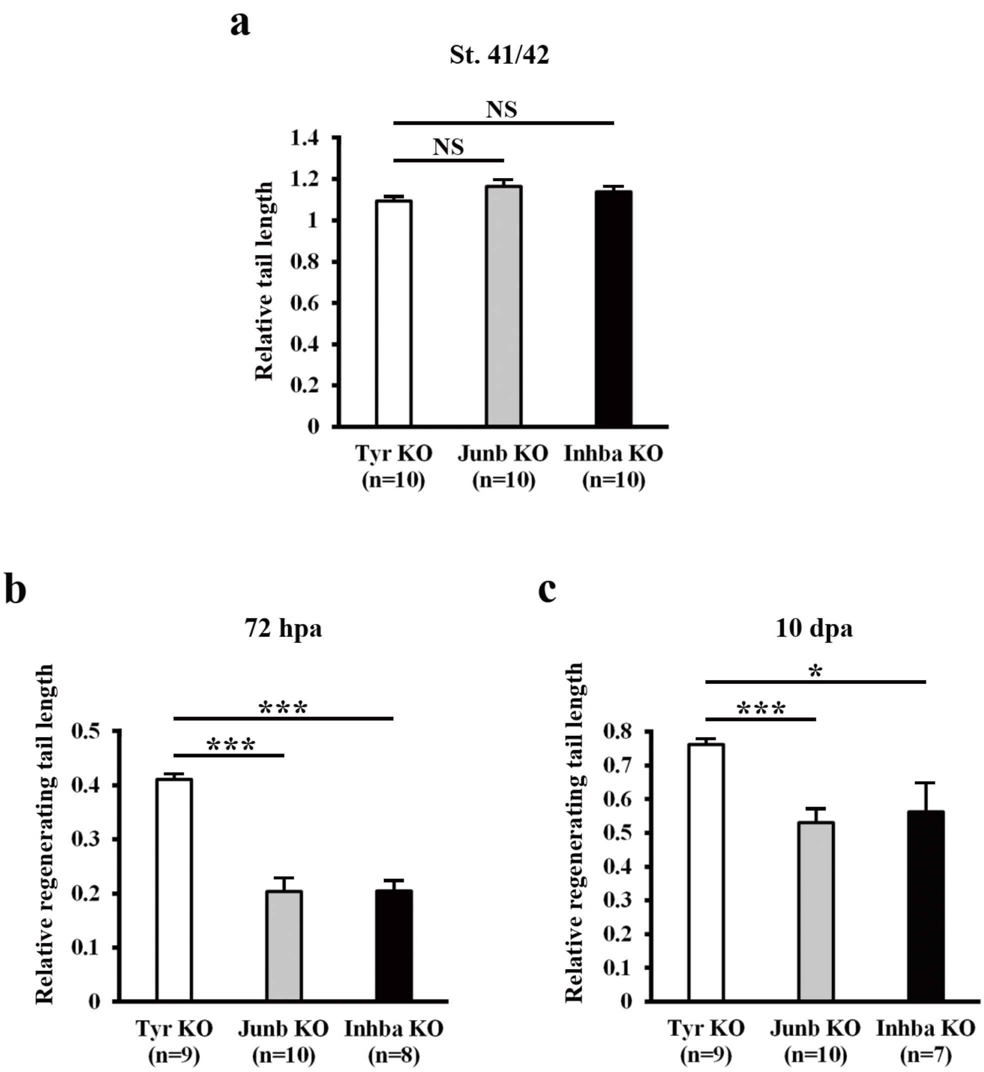

Supplementary Figure 7. *inhba* KO and *junb* KO affected tail regeneration but not embryonic tail development, and the regeneration defect was observed even at 10 dpa

Relative length of developing tadpole tails (the ratio of tail length to snout-to-vent body length) was calculated at stage 41/42 (**a**). After tail amputation, relative length of regenerating tails was evaluated at 72 hpa (**b**) and 10 dpa (**c**). NS, not significant; *P < 0.05, ***P < 0.001.

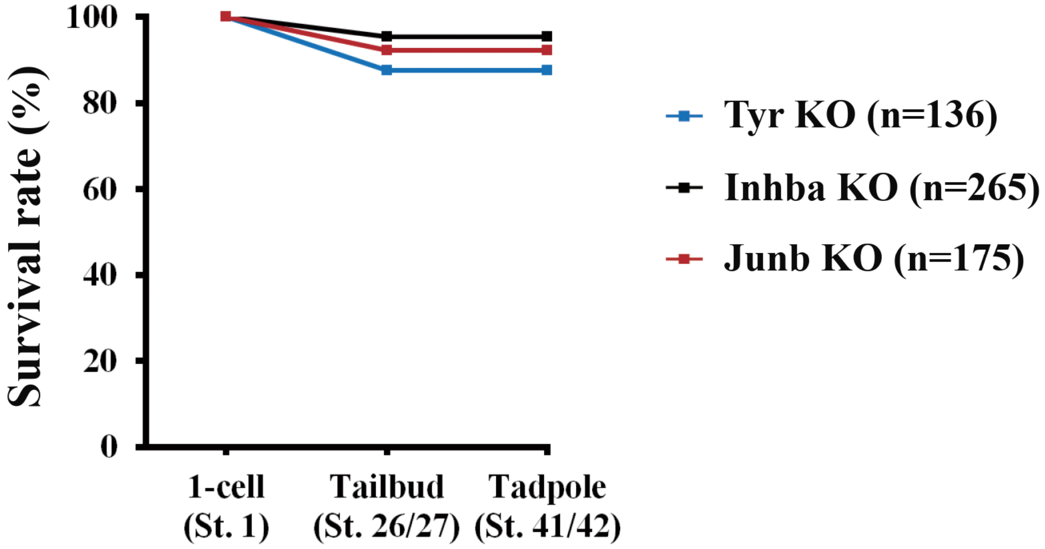

Supplementary Figure 8. Survival of KO embryos

The survival rate of *tyr* KO, *inhba* KO, and *junb* KO embryos was calculated at the three developmental stages shown in the figure.

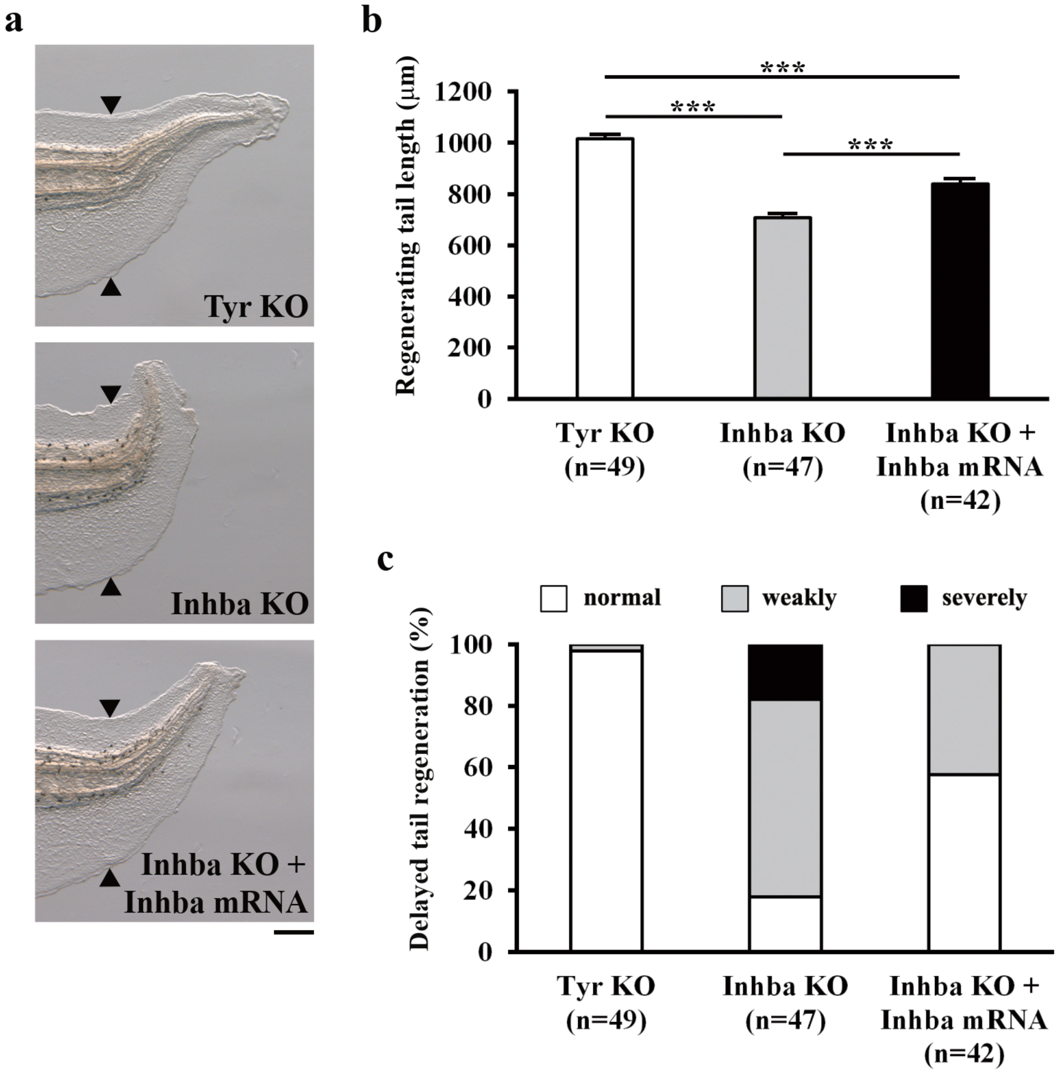

Supplementary Figure 9. Overexpression of InhibinβA rescued delayed tail regeneration in *inhba* KO tadpoles

(**a**) Representative phenotypes of *tyr* KO (control), *inhba* KO, and *inhba* KO + *inhba* mRNA tadpoles at 72 hpa. (**b**) The lengths of regenerating tails in KO tadpoles at 72 hpa. (**c**) Summary of phenotypes in KO tadpoles at 72 hpa. On the basis of the lengths of regenerating tails at 72 hpa, tadpoles were classified into three phenotypic groups (normal regeneration, weakly delayed regeneration, or severely delayed regeneration). Black arrowheads indicate amputation sites. Scale bar, 200 μm. ***$P < 0.001$.

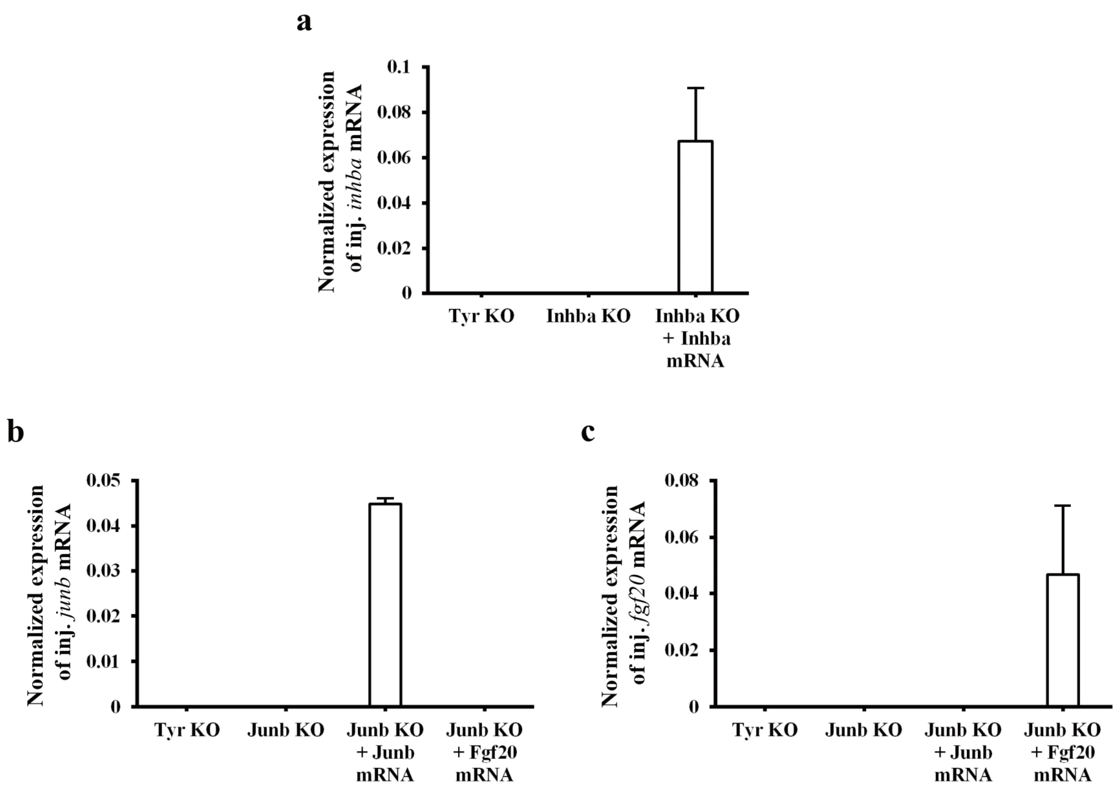

Supplementary Figure 10. Expression of injected mRNAs during tail regeneration

Expression of injected *inhba* (**a**), *junb* (**b**), and *fgf20* (**c**) mRNAs in regenerating tadpoles at 36 hpa was measured by qRT-PCR using the specific primer sets for each injected mRNA. The results show expression in arbitrary units normalized to the internal control *rps18*.

a

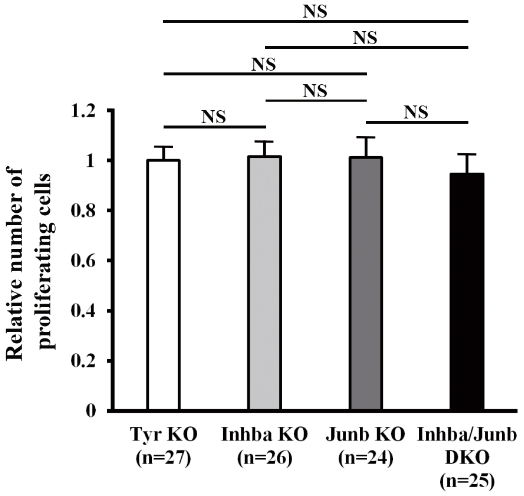

b

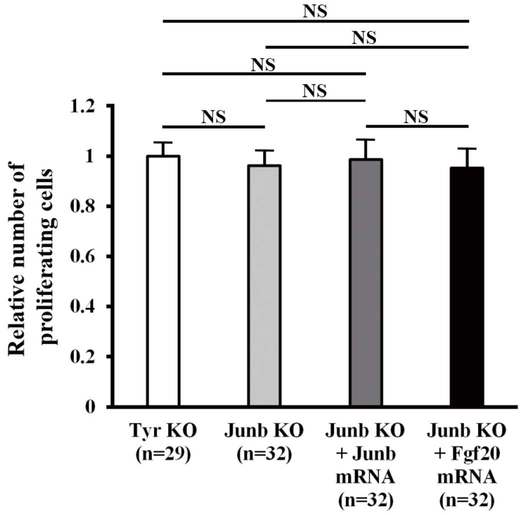

Supplementary Figure 11. Cell proliferation in the proximal tail region from amputation sites

Relative number of proliferating cells in the proximal region (extended 200 μm from amputation sites) in *tyr* KO (control), *inhba* KO, *junb* KO, *inhba*/*junb* DKO, *junb* KO + *junb* mRNA, and *junb* KO + *fgf20* mRNA tadpoles at 36 hpa. These data were obtained from the samples shown in Figure 4c (**a**) and Figure 5e (**b**). The number of pH3-positive cells was divided by the corresponding area. All values were normalized against the value of *tyr* KO. NS, not significant.

Uncropped gel image

Supplementary Figure 5

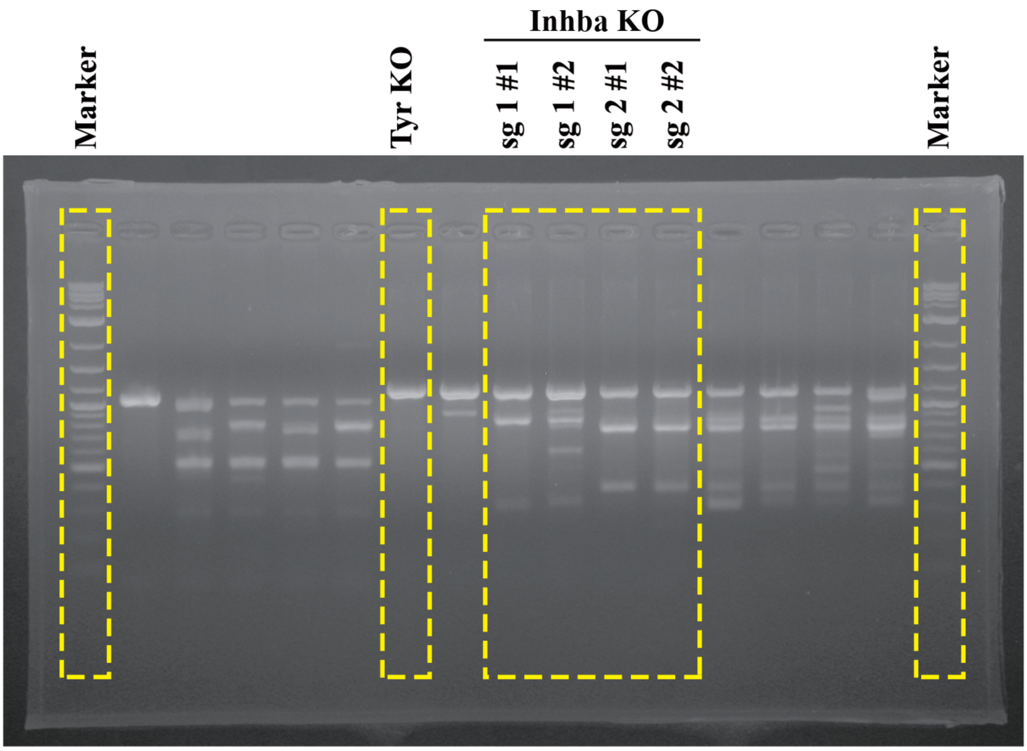

Supplement: Supplementary file 1 — Supplementary Information. [file 41598_2024_54280_MOESM1_ESM.pdf]
